# ADNP is associated with immune infiltration and radiosensitivity in hepatocellular carcinoma for predicting the prognosis

**DOI:** 10.1186/s12920-023-01592-x

**Published:** 2023-07-31

**Authors:** Xuan Wang, Honghua Peng, Ganghua Zhang, Zeyuan Li, Zhangyan Du, Bin Peng, Peiguo Cao

**Affiliations:** 1 Department of Oncology, Third Xiangya Hospital, Central South University, No.138 Tongzipo Road, Yuelu District, Changsha, 410013 Hunan People’s Republic of China; 2 Department of General Practice, Third Xiangya Hospital, Central South University, Changsha, 410013 Hunan People’s Republic of China

**Keywords:** ADNP, Prognosis, Hepatocellular carcinoma, Immune infiltration, Radiosensitivity

## Abstract

**Background:**

Hepatocellular carcinoma (HCC) is one of the most lethal diseases due to its high faculty of invasiveness and metastasis. Activity-dependent neuroprotective protein (ADNP) has been regarded as an oncogene in bladder cancer and ovarian cancer. However, the role of ADNP in the regulation of tumor immune response, development, and treatment resistance in HCC remains unknown and is worth exploring.

**Methods:**

The correlation between ADNP and prognosis, immune cell infiltration, immune checkpoints, chemokines, tumor mutation burden, microsatellite instability, and genomic mutation of pan-cancer cohorts in The Cancer Genome Atlas was analyzed. ADNP expression in HCC cell lines, HCC and the adjacent normal tissues was measured by western blotting and immunochemistry. Nomogram was constructed to predict the survival of patients with HCC based on the ADNP expression and significant clinical characteristics. The potential biological functions and impacts on radiotherapy of ADNP in HCC cell lines were verified by vitro experiments.

**Results:**

ADNP was upregulated in most cancers and patients with elevated ADNP expression were related to poor survival in several types of cancers including HCC. Functional enrichment analysis showed ADNP participated in the pathways correlated with coagulation cascades and DNA double strand break repair. Further, ADNP exhibited a negative correlation with the immune score, stromal score, estimated score, and chemokines, and a positive correlation with cancer-associated fibroblasts, myeloid-derived suppressor cells, neutrophils, regulatory T cells, and endothelial cells. Immunochemistry and western blotting results demonstrated ADNP was up-regulated in HCC. Vitro experiments verified that suppressing the ADNP expression significantly inhibited the proliferation, invasion and migration and elevated the radiosensitivity via decreasing DNA damage repair in HCC.

**Conclusion:**

ADNP might play an oncogene and immunosuppression role in tumor immune infiltration and response, thus influencing the prognosis. Its downregulation could attenuate the proliferation, invasion, migration, radioresistance of HCC. Our results indicated the potential of ADNP as a promising biomarker to predict the survival of HCC patients, providing a theoretical basis for novel integrative strategies.

**Supplementary Information:**

The online version contains supplementary material available at 10.1186/s12920-023-01592-x.

## Background

Activity-dependent neuroprotective protein (ADNP) gene is located in a chromosomal region, 20q12 [1] as a component of the ChAHP (CHD4-ADNP-HP1) complex [2], with certain functions in neurodegenerative diseases and tumors. As a highly conservative transcription factor, ADNP is overexpressed in the fetus, cerebellum, and cerebral cortex [3], participating in neuronal differentiation, neurite outgrowth [4], and neuroprotection with davunetide, a fragment peptide enhancing its activity. Besides that, research also indicated that ADNP was closely related to some kinds of neurological diseases including Parkinson’s disease [5], Alzheimer’s disease [6], and seizure [7].

Recently, exploring the character of ADNP in cancers has become a research hotspot. Recent work reported that ADNP acted as an oncogene in high-grade serous ovarian cancer (HGSOC) by accelerating the dysregulation of cell cycle checkpoints [8]. In addition, the overexpression of ADNP also facilitated the development of human bladder cancer via triggering the cell cycle transition process from the GI phase to the S phase through the AKT pathway [9]. In contrast to the results in HGSOC and human bladder cancer, ADNP functioned as a WNT repressor and exerted its anti-tumor impact on invasion, migration, and proliferation of colon adenocarcinoma (COAD) with an improved prognosis [10], which was consistent with the research in triple-negative breast cancer (TNBC) [11] and glioblastoma multiforme (GBM) [12]. Since the varied roles of ADNP were still controversial in the tumorigenesis and progression of multiple cancers, it was meaningful to further investigate the potential mechanism ADNP involved in hepatocellular carcinoma (HCC).

HCC is a kind of common cancer with a high mortality rate around the world [13]. Due to the presence of underlying liver dysfunction and a concomitant malignancy in the process of treatment-naive HCC, patients still have poor prognosis after standard treatment including sorafenib, radiofrequency ablation, vascular catheterization, surgical resection, and liver transplantation. Recently, radiation therapy and immunotherapy have achieved considerable advancements in HCC. IMbrave 150 clinical trial illustrated that combining atezolizumab with bevacizumab noticeably increased the median OS in comparison to sorafenib for unresectable HCC [14]. Meanwhile, a promising synthetical treatment, known as intensity-modulated radiotherapy combined with anti-angiogenic therapy plus immunotherapy, has also significantly proven the patients’ survival with advanced HCC [15]. Unfortunately, only a part of patients can benefit from the treatment accounting for low immune response and radioresistance. Therefore, it’s particularly important to explore more effective therapeutic biomarkers in HCC for classifying patients and choosing the best treatment plans.

Since ADNP was regarded as having a crucial cancer-promoting role in diverse kinds of tumors, its specific function in HCC remains unidentified. Hence, we conducted this comprehensive research and first reported ADNP might be a predicted biomarker and therapeutic target in HCC. The results of in vitro experiments also demonstrated its promoting impact on the proliferation, migration, invasion, and radioresistance in HCC cells.

## Methods

### Gene expression analysis

Based on the cancer genome atlas (TCGA), genotype tissue expression (GTEx), and Human Protein Atlas (HPA), we assessed the connection between ADNP and tumor samples. The expression of ADNP was evaluated by the website “http://vip.sangerbox.com/login.html”, which obtained ADNP expression data value transformed with Log_2_(x + 0.001) using UCSC Xena (https://xenabrowser.net/). Differences of ADNP expression value in tumor samples and normal samples were tested for significance using unpaired Wilcoxon rank-sum test and Wilcoxon signed-rank test. With the GEPIA database (http://gepia.cancer-pku.cn/), the difference in ADNP expression between tumor and normal tissue was compared and displayed by box plot. The expression of ADNP in distinct pathological stages was further determined simultaneously using the GEPIA website in pan-cancer. CPTAC database in the UALCAN website [16] (http://ualcan.path.uab.edu/cgi-bin/) was also applied for the comparison of tumor and normal tissue in total ADNP protein levels. The immunohistochemical (IHC) results were obtained from HPA [17] (https://www.proteinatlas.org/) to further assess ADNP protein levels.

### Survival prognosis analysis

To certify the prognostic value of ADNP, we conducted the univariate survival analysis of ADNP across pan-cancer through the Sanger-box website. A Cox proportional hazards regression model was constructed with the R software package “survival” (version 3.2.7). The overall survival (OS) and disease-specific survival (DSS) were also analyzed with the same software. An optimal cut-off value for ADNP was calculated by the R software package "maxstat". The minimum group sample size was more than 25%, and the maximum sample number was less than 75%. Based on the optimum cut-off value, the patients were separated into high- and low-expressed groups, and the survfit function profiling of the R software package “survival” was used to analyze the prognosis differences.

### Genomic alteration analysis

We acquired the genomic alteration rates (including mutation, structural variation, amplification, deep deletion and multiple alterations) of ADNP across cancers in the cBioPortal for cancer genomics website [18, 19] (http://www.cbioportal.org/). The “Mutation” module displayed the alteration rate using the bar plots and the mutation site position of ADNP visualized by stick figure and three-dimensional (3D) structure.

### ADNP related gene enrichment analysis

For further probing the molecular mechanism of ADNP in carcinogenesis, the Kyoto Encyclopedia of Genes and Genomes (KEGG), the Gene Ontology (GO), and the Gene Set Enrichment Analysis (GSEA) of ADNP were conducted in breast invasive carcinoma (BRCA), brain lower grade glioma (LGG), and HCC. First, we conducted differential expression analysis using the R package “DESeq2” in the high- and low-expressed groups, filtrating the differentially expressed genes (DEGs). Afterward, Go analysis was conducted of DEGs with the R package “cluster Profiler” [20] to explore the underlying biological function of ADNP, containing biological processes (BPs), molecular functions (MFs), cellular components (CCs) which defined as the location where molecular processes occur [21]. KEGG analysis [22, 23] was used to identify the significantly enriched metabolic pathways and signal transduction pathways [24]. Modification *p* < 0.05 was perceived as statistically significant. By comparing ADNP gene expression matrix in the high- and low-expressed groups via GSEA referring to c2.cp.kegg.v7.2.symbols.gmt, we explored the possible molecular mechanisms in signal pathways. FDR < 0.25, *p* < 0.05, and |NES|≥ 1 were considered statistically significant.

### Immune infiltration analysis

To visualize the statistical Spearman correlations between CD8 + T cells, macrophages, cancer-related fibroblasts (CAFs), endothelial cells, Tregs, myeloid-derived suppressor cells (MDSCs) and ADNP expression in pan-cancer, a heat map was generated by logging into the Tumor Immune Estimation Resource 2.0 (TIMER2.0) [25] (http://timer.cistrome.org/), with a positive relation in the red square (*p* < 0.05) and a negative relation in the purple square (*p* > 0.05). The relationship between ADNP expression and immune cells in HCC was further presented by scatter diagram. Sanger-box was used to further analyze immune infiltrations across distinct cancers. Related expression data of ADNP was downloaded from UCSC Xena dataset, then the ADNP expression profiles were extracted and were mapped to the corresponding gene symbol. The R package “ESTIMATE” was used to calculate the immune score, stromal score, and estimate score in each tumor sample and then the correlation was evaluated by calculating the Pearson correlation coefficient using R package “psych”. We enunciated the relationship between ADNP and immunomodulators encompassing immune stimulators and inhibitors, chemokines as well as major histocompatibility complex by the TISIDB website [26] (http://cis.hku.hk/TISIDB/index.php). Using the Sanger-box, we further investigated the statistical Pearson relation between ADNP expression and tumor mutation burden (TMB) and microsatellite instability (MSI), which was calculated by the R package “maftools” (version 2.8.05).

### Clinical features related analysis of patients with HCC

R package (version 4.1.1) was used to indicate the correlation between ADNP expression and T stage, pathologic stage, histological grade, and AFP expression levels. Nomogram, a prediction model including T stage, M stage, pathologic stage, and ADNP expression level, was constructed with the univariate and multivariate analysis to estimate the survival of HCC patients at one, three, and five years by R package “rms” and “survival”. Nomogram performance and discrimination were evaluated by concordance index (C-index), area under the curve (AUC), and calibration curve.

### Immunohistochemistry analysis

The Department of Pathology, Third Xiangya Hospital, Central South University furnished eight pairs of human patient paraffin sections of HCC tissue specimens and the adjacent peritumoral tissue specimens. After dewaxed with xylene, the paraffin sections were rehydrated with absolute ethanol, heated in citric acid buffer (PH 6.0) for repairing the antigens, then cooled and washed in PBS (PH 7.4) three times for 10 min. After set in 3% H_2_O_2_ solution for blocking endogenous peroxidase, the paraffin sections were blocked with 3% BSA at room temperature for 30 min, bound with anti-ADNP (Abcam, ab300114, 1:50) overnight at 4 ℃, and incubated with secondary antibody (Immunoway, RS002, Beijing, China) at room temperature for 50 min. Finally, the DAB kit (Solarbio, DA1010, Beijing, China) was performed for color development, and 3D Histech Pannoramic Scan (3D Histech, Pannoramic Scan, Hungary) was used for scanning. Two independent pathologists conducted IHC staining scoring referring to the previous study [9]. The staining index, an assessment method for evaluating ADNP expression in HCC and normal liver tissue, was obtained from the multiplication of the scores graded by percentage positive cells and staining power with scores ≥ 6 considered as high expression and < 6 considered as low expression.

### Cell lines and cell culture

American Type Culture Collection (ATCC, Manassas) provided the original human HCC cell lines (Hep 3B, SMMC-7721, HCCLM3, Huh7), which were cultured in DMEM (BasalMedia, L510KJ, Shanghai, China) containing 10% fetal bovine serum (FBS), 100U/ml penicillin, and 100 g/ml streptomycin with 37 ℃ and 5% CO2 in a humidified incubator. All experiments were completed in the mycoplasma-free condition.

### Western blotting analysis

Hep 3B, SMMC-7721, HCCLM3, and Huh7 cells were treated with PMSF(1 mM) and then placed on ice, incubated for 20 min, and centrifuged at 13,000 rpm for 20 min at 4 ℃. The supernatants were collected, and the total protein was extracted by RIPA lysis buffer (TDY Biotech Company, WB0003, Beijing, China). Then BCA protein assay kit (TDY Biotech Company, WB0028, Beijing, China) was conducted to detect the protein content. Afterward, the protein concentration of the lysates was adjusted to 4 mg/ml with RIPA, then the lysates were heated with 5xSDS buffer solution (TDY Biotech Company, WB0031, Beijing, China) for 5 min, separated on 10% SDS–polyacrylamide gels (Sigma, L4390, America), and transferred to NC membranes (Millipore, HATF00010, America) for 1 h. The membranes were blocked with 3% BSA-TBST for 30 min and incubated together with the primary ADNP (Abcam, Ab300114, America), γ-H2AX (Abcam, ab81299, America) and β-actin (Immunoway, YM3029, America) antibodies diluted with 3% BSA-TBST (1:1000) overnight at 4 ℃. After washed with TBST buffer for 3 min five times, the membranes were incubated with a corresponding secondary antibody [anti-rabbit or anti-mouse IgG (H + L) biotinylated antibody (TDY Biotech Company, S004/S001, Beijing, China)] diluted by 5% skimmed milk powder (1:10,000) for 40 min at room temperature. Then ECL reagents (Millipore, WBkls0500, America) were used to visualize the protein-antibody bound bands after washing the membranes with TBST buffer for 3 min six times. The IOD value was gotten by MultiSkan3 microplate reader, quantified with ImageJ software, and normalized with β-actin. Histograms were conducted by GraphPad Prism 9.0 software to visualize the results.

### Cell transfection

Hep 3B and HCCLM3 were selected for transfection to conduct the functional experiment, which was seeded onto a 6-well plate with 1 × 10^5^/ml density to achieve an 80% cell confluency while conducting small interfering(si) RNA transfection. The targeting sequence used for siRNA against ADNP was siRNA-2944: sense (5’-3’) GAAGAAGAAUCCAAUGAAATT, antisense (5’-3’) UUUCAUUGGAUUCUUCUUCTT; siRNA-1763: sense (5’-3’) GCAAAUGCCUCUACUGUAATT, antisense (5’-3’) UUACAGUAGAGGCAUUUGCTT; siRNA-3531 sense (5’-3’) CAACAUGACUGAUGGAGUATT, antisense (5’-3’) UACUCCAUCAGUCAUGUUGTT (GENERAL BLOL, Anhui, China). After diluted with opti-MEM culture medium (GIBCO 31985070, America), siRNAs were mixed with the Lipofectamine 3000 (Invitrogen, L3000-015, America) at room temperature for 20 min. After cultured in a CO_2_ incubator for 24 or 48 h, the cells were adopted for the follow-up experiments. Real-time quantitative polymerase chain reaction (RT-qPCR) was performed to examine ADNP knockdown efficiency.

### RT-qPCR

Relative ADNP expression was verified by RT-qPCR in the transfected cells. The primer sequences for ADNP amplification were as follows: forward: 5’-CATCACTTACGAAAAACCAGGACTA-3’; reverse:5’-TGCTGAGGCTGCTACTTGGT-3’. Each group carried out three repetitions. The cDNA of ADNP was synthesized by Revert Aid First Strand cDNA Synthesis Kit (ThermoFisher, K1622, America) following the protocol. RT-qPCR was conducted using 5uL 2xSuperReal PreMix Plus (SYBR Green, TianGen, FP205-02, Beijing, China), 0.3uL primer mix(10uM), 0.5uL cDNA and 4.2uL H_2_O on Real-Time PCR Detection System (LightCycler480, America) according to the manual. ADNP expression was calculated using 2-△△Ct with normalization to the normal control (β-actin).

### Irradiation

Hep 3B and HCCLM3 cell lines were irradiated with 6MV high energy X-ray by a linear accelerator (Varian company, Unique, American) at 400 MU/min, screened by vertical radiation at five different gradients of 0, 2, 4, 6, 8 Gy and source axle distance of 100 cm.

### Cell counting kit8 (CCK8) cell viability assay

After transfected with si-ADNP for 24 h, the Hep 3B/HCCLM3 cells were collected and seeded in 96-well plates with the density of 5 × 10^3^ cells/well at 37 ℃ with 5% CO_2_. 100ul CCK8 solution (Beyotime, C0039, Shanghai, China) was added to each well, after which the cells were incubated for 4 h. The optical densities at 450 nm were measured with a microplate reader (Biotek, Elx808, America) to determine the proliferation ability of cancer cells.

The Hep 3B/HCCLM3 cells were inoculated into 36-well plates of 5 × 10^4^ and 6.5 × 10^4^ cell/well, transfected with si-ADNP for 24 h, exposed to vertical radiation (0, 2, 4, 6, 8 Gy) and cultured in an incubator. Then 100 ul/well CCK8 solution was added per well at 24, 48, and 72 h. Other details were accomplished as previously mentioned.

### Transwell invasion and migration assay

Following transfection for 48 h, the Hep 3B/HCCLM3 cells were collected. 100ul matrigel was thawed overnight at 4 ˚C in a refrigerator and diluted in 700ul serum-free medium. 100ul matrigel was added into the upper transwell chamber (Costar, 3422, America) which was allowed to solidify for 2 h at 37℃. The transfected cells (5 × 10^5^) were inoculated into the upper chamber, and a total of 500ul medium containing 15% FBS was added to the low chamber. Following incubation at 37 ℃ with 5% CO_2_ for 48 h, the cells and matrigel on the surface of the upper chamber were wiped. The transwell chamber was washed three times with PBS, fixed with 4% paraformaldehyde for 20 min at room temperature, washed three times with PBS again, and stained with 0.1% crystal violet for 15 min. The transwell chamber was then observed, photographed, and counted with a microscope (Motic, AE31, Xiamen, China). The migration experiment process was terminated at 24 h, and the upper chamber was cultured without matrigel, else by the foregoing cell invasion assay.

### Colony formation assay

The HCC cells were transfected for 24 h, collected, and seeded into a 35 mm cell culture dish with 1000 cells of single-cell suspension per dish. Every group included 3 dishes. Following 2 weeks of cell incubation at 37 ℃ with 5% CO_2_, the culture was terminated with colonies visible to the naked eye. The culture supernatant was discarded, and the cells were washed with PBS three times and fixed with 1 ml paraformaldehyde for 15 min. The fixative solution was discarded, and the cells were stained with 1% crystal violet dye solution (Solarbio, C8470, Beijing, China) for 10 min at room temperature, washed with PBS three times, and detected under the microscope. Divided seeded cells into clone amounts obtained the cloning efficiency. Each colony consisting of more than 50 cells was counted as a positive colony.

### Immunofluorescence (IF) staining

IF was performed on Hep 3B cells and HCCLM3 cells with 4 Gy irradiation to detect the γ-H2AX expression level. The cells were incubated with anti-γ-H2AX (Abcam, ab81299, America, 1:250) at 4 ℃ overnight after the standard process, which was followed by the incubation with the second antibody against ADNP (Abcam, ab300114, America, 1:1000) for 1 h at 37 ℃, stained with DAPI (10 min, room temperature) to display the nucleus. Fluorescence images were further captured by a fluorescence microscope (Olympus, CX23, Japan).

### Statistical analysis

Unpaired Wilcoxon rank sum and signed rank tests were utilized for the comparison of ADNP expression between the tumor and normal tissues. The log-rank test method was used to evaluate the significant differences in prognosis between the high- and low-expressed groups, and then the Kaplan–Meier survival plots were generated. For correlations, Pearson or Spearman correlations were performed. Student’s t-test was used to analyze the differences between all the paired groups in the research. All experiment data were analyzed by GraphPad Prism 9.0 software. Throughout the text, *p* < 0.05 was examined to be statistically significant. (*p < 0.05, **p < 0.01, ***p < 0.001, ****p < 0.0001).

## Results

### ADNP was expressed differently and exhibited prognostic value in pan-cancer

**Fig. 1:**
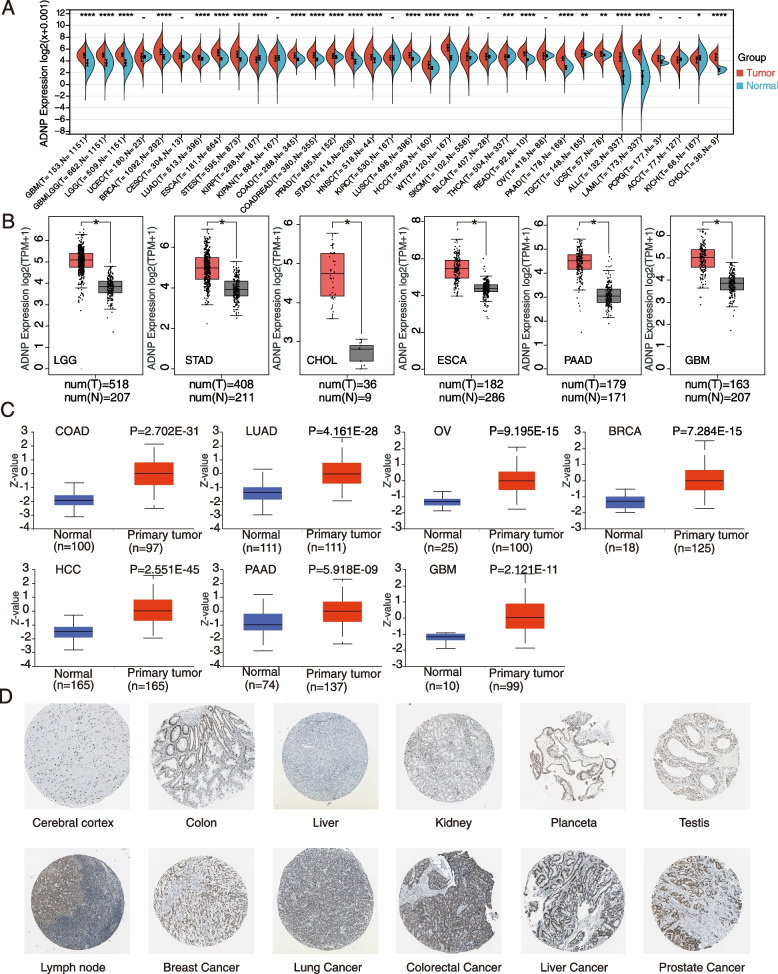
ADNP expression levels in different types of human cancers. **A** Comparisons of ADNP expression levels between tumor tissues from TCGA database and normal tissues from GTEx database. **B** Box plot data of LGG, STAD, CHOL, ESCA, PAAD, GBM in TCGA database compared to normal tissues in GTEx database. **C** The box plot shows ADNP protein differences between normal tissue and primary tumor in COAD, LUAD, OV, BRCA, HCC, PAAD, GBM. **D** Representative IHC images of ADNP expression in normal cerebral cortex tissues, normal colon tissues, normal liver tissues, normal kidney tissues, normal placenta tissues, normal testis tissues, normal lymph node tissues, breast cancer, lung cancer, colorectal cancer, liver cancer, prostate cancer

As shown in Fig. 1A, the ADNP expression levels in GBM, LGG, BRCA, cholangiocarcinoma (CHOL), COAD, esophageal carcinoma (ESCA), HCC, stomach and esophageal carcinoma (STES), lung adenocarcinoma (LUAD), colon adenocarcinoma/rectum adenocarcinoma (COADREAD), acute myeloid leukemia (AML), pancreatic adenocarcinoma (PAAD), prostate adenocarcinoma (PRAD), stomach adenocarcinoma (STAD), skin cutaneous melanoma (SKCM), head and neck squamous cell carcinoma (HNSC), high-risk Wilms tumor (WT), testicular germ cell tumors, uterine carcinosarcoma (UCS), rectum adenocarcinoma (READ), thyroid carcinoma (THCA), and acute lymphoblastic leukemia (ALL) (*p* < 0.05) were statistically higher than that in the corresponding normal tissues. While in kidney renal papillary cell carcinoma and kidney chromophobe, the condition was the opposite. We further displayed the ADNP expression differences between the tumor and normal tissues in CHOL, ESCA, GBM, LGG, PAAD, and STAD using GEPIA database (Fig. 1B). Based on CPTAC dataset, ADNP protein expression was statistically higher in BRCA, HCC, LUAD, GBM, ovarian serous cyst adenocarcinoma (OV), PAAD, and COAD (Fig. 1C). Comparing the homologous normal tissues with carcinoma tissues in HCC and COAD, the IHC results intuitively revealed a significant upregulation of ADNP protein expression (Fig. 1D).

**Fig. 2:**
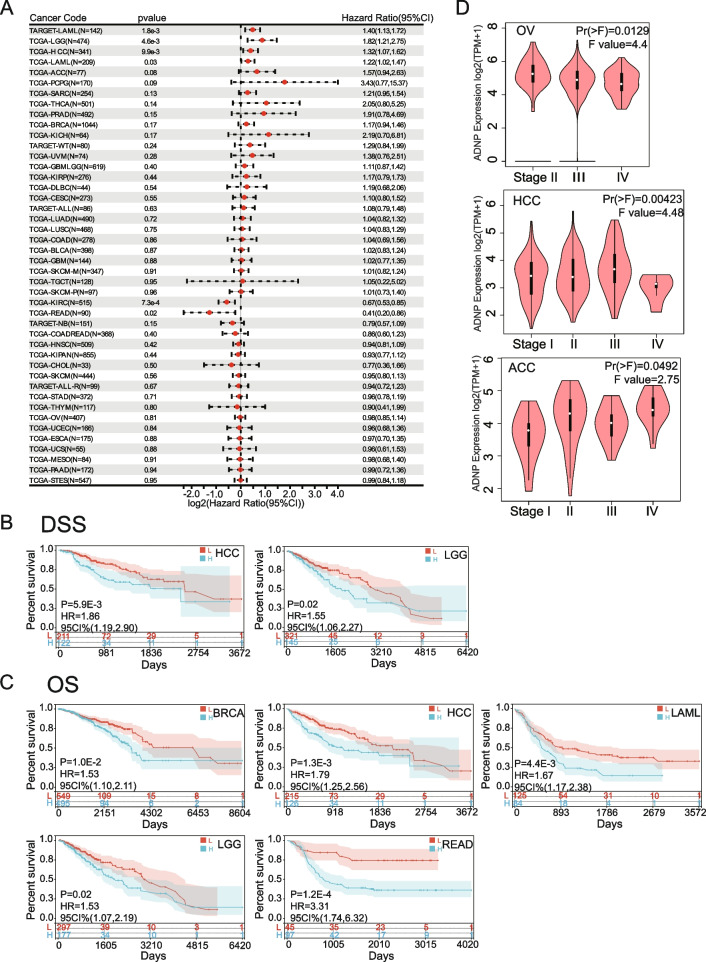
Survival analysis comparing the high and low expression of ADNP in different types of cancer. **A** Relation between ADNP expression and patient prognosis (OS) of different cancers in TCGA database. **B** Survival curves of disease-specific survival (DSS) in two cancer types (LGG, HCC). **C** Survival curves of OS in six cancer types (BRCA, LAML, LGG, HCC, READ). **D** Violin plots present ADNP expression levels in different pathological stages in OV, HCC, ACC with statistically significant differences

The Survival analysis was depicted in the forest map (Fig. 2A). We found that the higher ADNP expression was correlated with the worse DSS in LGG (*p* = 0.02, 95%CI: 1.06–2.27, HR = 1.86), HCC (*p* = 0.0059, 95%CI: 1.19–2.90, HR = 1.55) (Fig. 2B). The statistically significant increase of high ADNP expression was related to worse OS for LGG (*p* = 0.02, 95% CI: 1.07–2.19, HR = 1.53), HCC (*p* = 0.0013, 95% CI: 1.25–2.56, HR = 1.79), LAML (*p* = 0.0044, 95%CI: 1.17–2.38, HR = 1.67), READ (*p* = 0.0001, 95% CI: 1.74–6.32, HR = 3.31), BRCA (*p* = 0.01, 95% CI: 1.10–2.11, HR = 1.53) (Fig. 2C).

To further discuss the underlying impact of ADNP expression on carcinoma progression and metastasis, we detected the ADNP expression levels in different pathological stages and observed a statistically significant difference in HCC, adrenocortical carcinoma (ACC), and OV. There was an increasing tendency of ADNP expression along with the pathological stages in HCC and ACC, conversely, a decreasing tendency in OV (Fig. 2D). Based on the above analysis, ADNP seemed to be a risk factor for LGG, HCC, LAML, READ, and BRCA.

### Analysis of genomic mutation

**Fig. 3:**
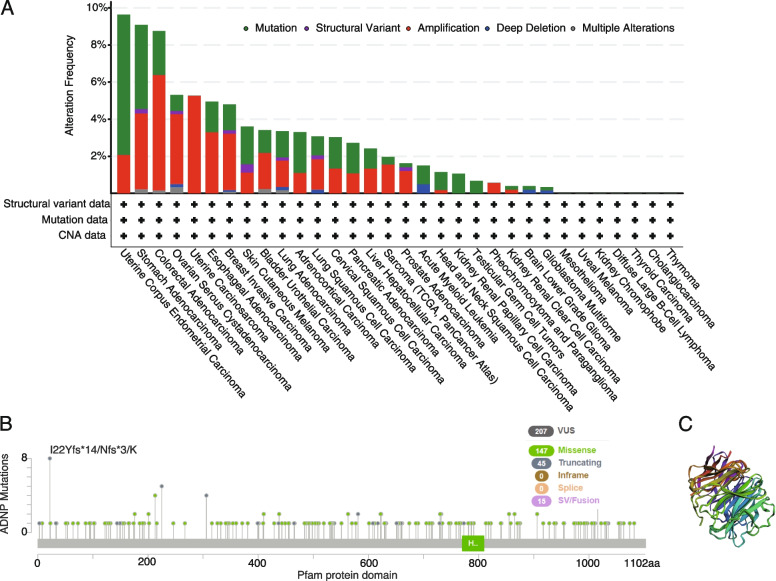
ADNP mutation landscape. **A** ADNP mutation frequency in multiple TCGA pan cancer studies using cBioPortal database. **B** Mutation diagram of ADNP in different cancer types across protein domains. **C** 3D structure presented the mutation types and the position of mutation sites

Genomic mutation analysis exhibited the sites, types, and case numbers of the ADNP mutations utilizing TCGA. We observed the total alteration frequencies were lower than 5% in most cancer types except for uterine corpus endometrial carcinoma (UCEC) (9.64% of 524 cases), STAD (9.09% of 440 cases), COAD (8.75% of 594 cases), OV (5.31% of 584 cases), UCS (5.26% of 57 cases). The amplification frequencies were high in UCEC (2.08%), STAD (4.09%), COAD (6.23%), OV (3.77%), UCS (5.26%), ESCA (3.3%), BRCA (3.04%) (Fig. 3A). We further explored the somatic mutation in pan-cancer, and found 207 mutations including 147 mistence,45 truncated mutations, and 15 fusion mutations (Fig. 3B). Different mutation types and the position of mutation sites were shown in the 3D structure (Fig. 3C).

### Enrichment analysis of ADNP related pathway

**Fig. 4:**
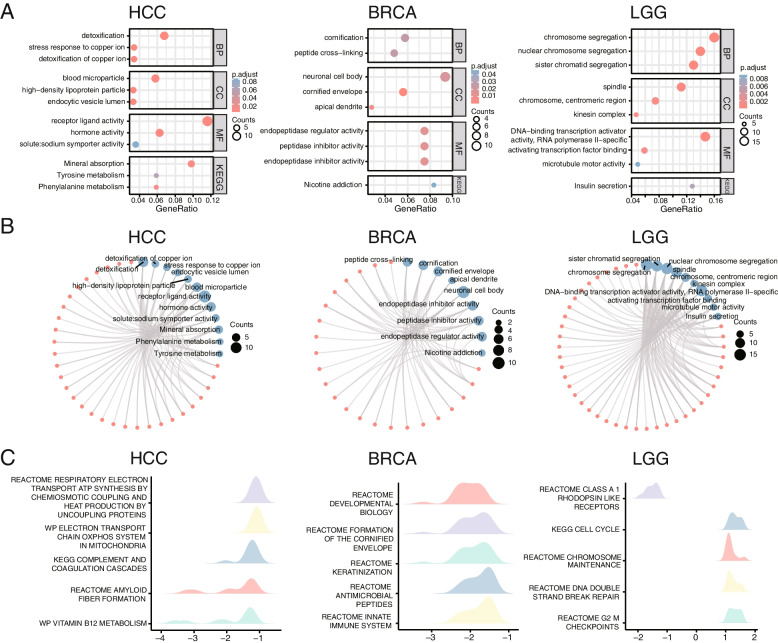
ADNP expression and function profiles in three representative cancers. **A-B** Go enrichment analysis in BPs, MFs, CCs and KEGG enrichment analysis exhibiting KEGG pathways of ADNP in HCC, BRCA, LGG. **C** Top 5 reactome pathways from GSEA in HCC, BRCA, LGG

With the comparison of the high- and low-expressed groups of ADNP, we identified ADNP-related DEGs, then the GO and KEGG enrichment analysis was conducted in BRCA, LGG, and HCC. The involved BPs were significantly enriched in the detoxification of copper ions, chromosome, nuclear chromosome segregation, sister chromatid segregation, detoxification, the stress response to copper ions, cornification, and peptide cross-linking. MFs included DNA-binding transcription activator activity, RNA polymerase II-specific, activating transcription factor binding, receptor-ligand activity, hormone activity, endopeptidase regulator or inhibitor activity, and peptidase inhibitor activity. In CCs, ADNP-related DEGs were mainly enriched in the spindle, chromosome, centromeric region, blood microparticles, high-density lipoprotein particles, endocytic vesicle lumen, neuronal cell body, and comified envelope (Fig. 4A). KEGG enrichment analysis manifested that ADNP-related DEGs were highly correlated with mineral absorption, phenylalanine metabolism, and insulin secretion preponderance (Fig. 4B). GSEA of ADNP-related DEGs was significantly enriched in Reactome pathways such as respiratory electron transportation, ATP synthesis, antimicrobial peptides, innate immune system, and DNA break repair with significant enrichment in KEGG pathways including cell cycle, complement, and coagulation cascades (Fig. 4C).

### Immune infiltration and immunotherapy biomarker analysis

**Fig. 5:**
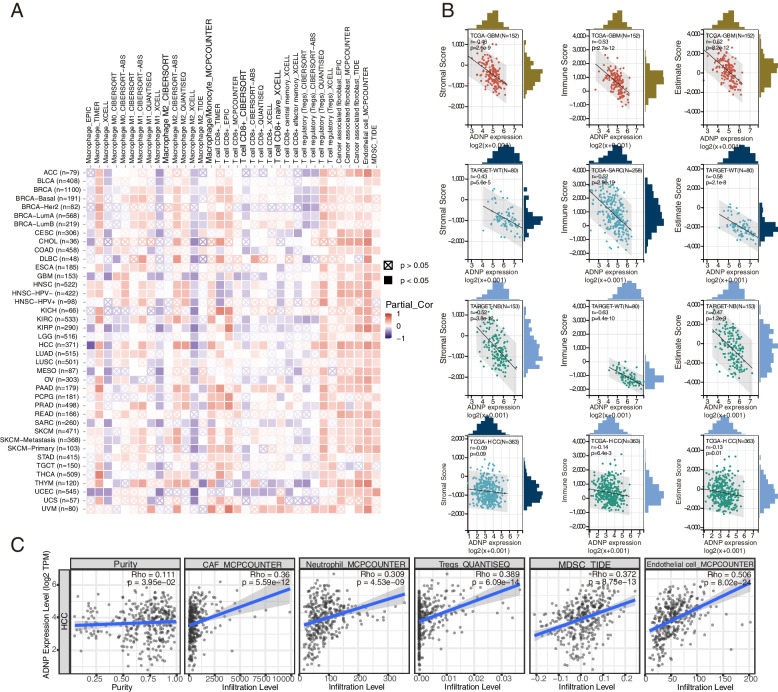
Correlations between ADNP and immune infiltration and immune cells. **A** Correlation of ADNP expression with six infiltrating immune cells (CD8 + T cells, macrophages, cancer-related fibroblasts, Tregs, endothelial cells, MDSC) in the TIMER 2.0 database. **B** Correlation of ADNP expression with immune score, stromal score and estimate score in pan cancer. The top three places and scores of HCC were displayed. **C** Correlations of ADNP expression with tumor purity and infiltrating immune cells including CAFs, endothelial cells, MDSC, Tregs, and neutrophils of HCC in the TIMER 2.0 database

In our research, we exhibited the correlation between CD8 + T cells, macrophages, CAFs, Tregs, endothelial cells, MDSCs and ADNP expression in various cancers of TCGA with a heat map. We observed ADNP expression had the most noteworthy positive correlation with the infiltration of CAFs, endothelial cells, and MDSCs in the majority of pan-cancers (Fig. 5A). We further studied the connection between ADNP expression and immune score, stromal score, and estimated score in pan-cancers using sanger-box. The results revealed ADNP was negatively associated with the stromal score in GBM (*r* = -0.46), WT (*r* = -0.43), and neuroblastoma (NBL) (*r* = -0.52). ADNP expression in GBM (*r* = -0.53), sarcoma (SARC) (*r* = -0.52), WT (*r* = -0.63) was most negatively related to immune score, and expression in GBM (*r* = -0.52), WT (*r* = -0.58), NBL (*r* = -0.47) was most negatively related to estimated score (Fig. 5B). Meanwhile, ADNP expression was negatively associated with immune score (*r* = -0.14) and estimated score (*r* = -0.13) in HCC. The details of the mentioned three scores in other cancer types were described in Table S1-3. Intriguingly, as results displayed in Fig. 5C, ADNP was positively related to CAFs (*r* = 0.36), MDSCs (*r* = 0.372), neutrophils (*r* = 0.309), Tregs (*r* = 0.389), and endothelial cells (*r* = 0.506) in HCC. We further showed the negative relationship between ADNP expression and chemokines as well as immunomodulators (Fig. S1A-D). Synthesizing all analyses, we drew a conclusion that ADNP might play an immune-evasive role in the tumor microenvironment (TME).

It was discovered that ADNP expression was significantly related to TMB in LUAD, COAD, STES, THCA, and COADREAD (Fig. S1E). ADNP expression also had a remarkable connection with MSI in lymphoid neoplasm diffuse large B cell lymphoma (DCLBCL), COAD, COADREAD, HNSC, PRAD, THCA, STES, BRAC, lung squamous cell carcinoma (LUSC), LUAD (Fig. S1F). These results revealed ADNP expression was strongly related to TMB and MSI, suggesting that ADNP might act as an active role to predict the immunotherapy of cancers.

### ADNP expression was related to the clinical features of the HCC patients

**Fig. 6:**
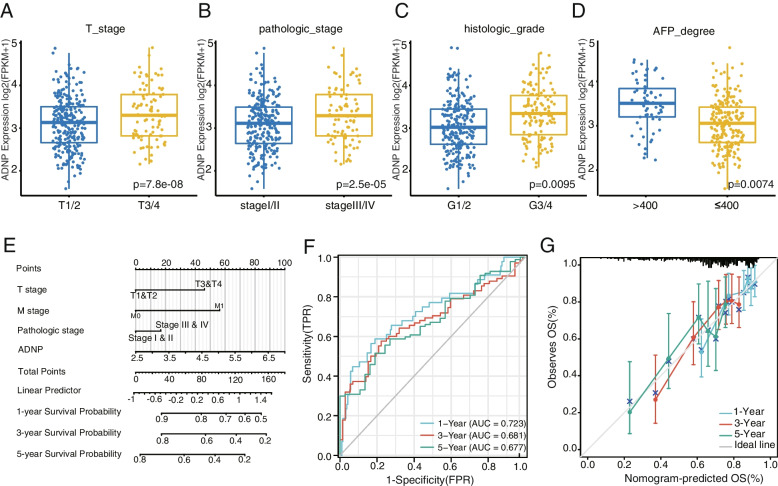
ADNP expression and clinical features correlation analysis in hepatocellular carcinoma. **A** The correlations between ADNP expression and T stage,** B** pathologic stage, **(C)** histological grade and **D** AFP expression levels. **E** Nomogram based on the ADNP expression and clinical information to predict the 1-, 3- and 5-year OS in hepatocellular carcinoma patients. **F** the prognosis evaluation of ADNP in 1 year, 2 years, and 5 years survival rate of hepatocellular carcinoma patients by ROC curve. **G** The calibration validated the consistency of predicted and real 1-, 3-, and 5-year OS outcomes

The foregoing bioinformatics analysis indicated ADNP might participate in the development of HCC. Hence, we examined the association between ADNP expression and vital clinical features of HCC patients. We found that elucidated the expression of ADNP was positively correlated with the higher T stage (Fig. 6A), pathologic stage (Fig. 6B), histological grade (Fig. 6C), and AFP expression (Fig. 6D), which indicated ADNP had an affinity with malignant progression and poor prognosis in HCC. A nomogram was constructed containing the T stage, M stage, pathological stage, and ADNP expression based on the Cox regression analysis (Fig. 6E). Meanwhile, the predictive ability of nomography was evaluated by 1 year (AUC = 0.723), 3 years (AUC = 0.681), and 5 years (AUC = 0.677) receiver operator characteristic (ROC) curve (Fig. 6F), with C-index = 0.713. As shown in Fig. 6G, the nomography had good accuracy.

### ADNP expression was up-regulated in HCC and associated with the proliferation, migration, and invasion

**Fig. 7:**
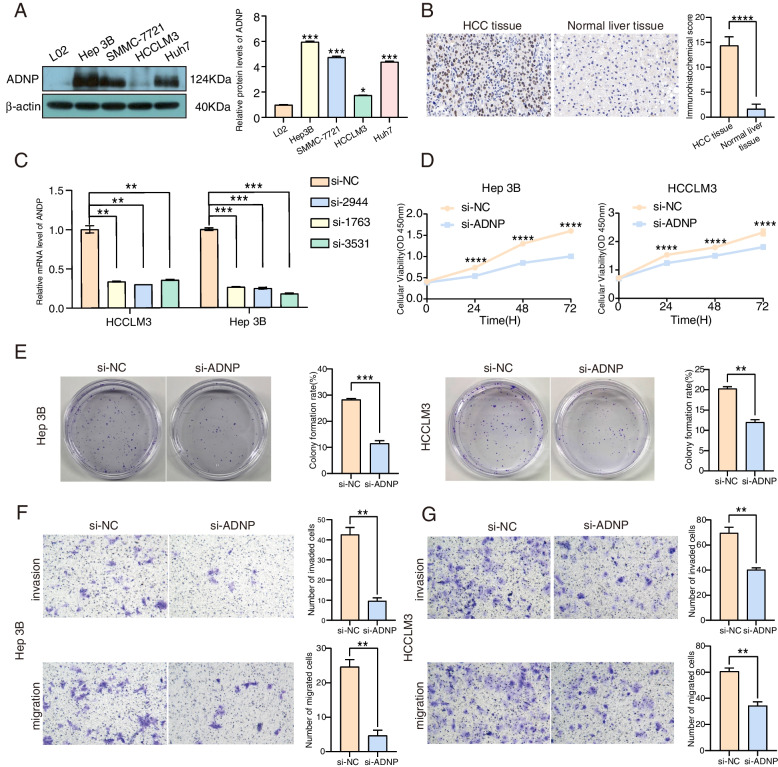
The verification of ADNP expression in tissue specimens and cell lines of hepatocellular carcinoma. **A** Expression levels of ADNP protein in HCC cell lines verified by western blotting. **B** IHC analysis of ADNP in patients with HCC. **C** RT-qPCR proved the efficiency of ADNP knockdown in Hep 3B and HCCLM3 cells. **D** ADNP knockdown inhibited cell proliferation detected in Hep 3B and HCCLM3 cells by CCK8 assay. **E** Colony formation assay was used to determine the hepatocellular carcinoma cell proliferation. **F-G** Transwell assay was performed to detect the cell invasion and migration of Hep 3B and HCCLM3 cells

As exhibited in Fig. 7A, ADNP protein in HCC cell lines (Hep 3B, SMMC-7221, HCCLM3, Huh7) showed enhanced expression levels compared to the normal liver cells. Correspondingly, we also verified higher expression of ADNP in the HCC tissues compared with the normal liver tissues with immunohistochemistry (Fig. 7B). To further investigate the effect of ADNP on HCC cells’ proliferation, invasion, and migration, the expression of ADNP in HCCLM3 and Hep 3B cells was successfully knocked down with the specific siRNAs (Fig. 7C). In the two HCC cell lines, the silenced ADNP groups both showed inhibited cell viability compared to the control groups by CCK8 assay (Fig. 7D). In the clonal formation experiments, we also demonstrated that the knock-down of ADNP significantly led to the restrained HCC cells’ proliferation (Fig. 7E). ADNP was also proved to be positively connected with the invasion and migration abilities of Hep 3B cell (Fig. 7F) and HCCLM3 cell (Fig. 7G) in the transwell experiment.

### Downregulated ADNP expression enhanced the radiosensitivity of HCC cells

**Fig. 8:**
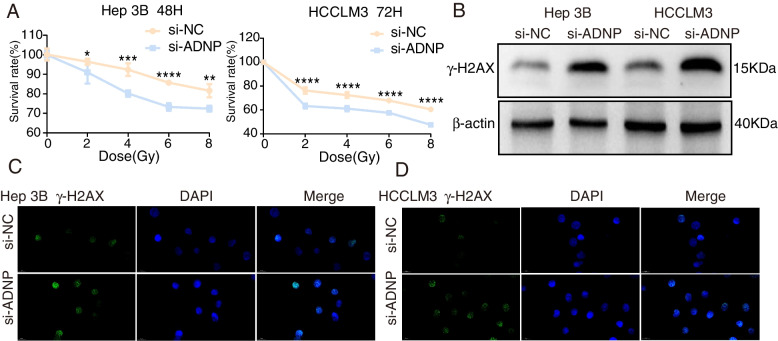
Impact of ADNP on radiosensitivity of HCC. **A** CCK8 assay verified impact of ADNP on radiosensitivity in Hep 3B cells and HCCLM3 cells. **B**The quantitative analysis of γ-H2AX by western blotting. **C-D** Representative IF staining images of γ-H2AX (red) and DHX9 (green) in Hep 3B cells and HCCLM3 cells

At 24 h after transfected with si-ADNP, Hep 3B and HCCLM3 cells were X-rayed with gradually elevated doses, respectively (0, 2, 4, 6, 8 Gy). The transfection efficacy of si-ADNP was detected by RT-qPCR. The radiosensitizing impacts on Hep 3B and HCCLM3 cells with or without the downregulation of ADNP were evaluated by CCK8 assays. Exposed to the same dose of irradiation, the survival percentage of cells with downregulation of ADNP significantly declined contrasted to the non-transfected ones as the control (Fig. 8A). Particularly, the difference between the ADNP knockdown and control groups in HCCLM3 cells was most apparent under 8 Gy ionizing radiation, while in Hep 3B cells the most different condition was under 6 Gy ionizing radiation.

Meanwhile, the influence of down-regulated ADNP expression on the radiosensitivity of HCC cells was further verified using IF Staining. Phosphorylated histone H2AX (γ-H2AX), a prognostic indicator for radiosensitivity, was involved in the cellular response to DNA double-strand breaks and facilitated post-replicational DNA repair [27]. Quantitative analysis of γ-H2AX was performed by western blotting (Fig. 8B). γ-H2AX foci were significantly increased in the ADNP knockdown groups of Hep 3B (Fig. 8C) and HCCLM3 cells (Fig. 8D)while conducted with 4 Gy irradiation. To sum up, we found that the downregulation of ADNP might influence the radiosensitivity of HCC cell lines by restraining the radiation-induced DNA damage repair.

## Discussion

In this study, we found the expression of ADNP was promoted in most cancers and associated with poor prognosis in LGG, HCC, LAML, READ, and BRCA. The expression of ADNP was positively related to CAFs, MDSCs, endothelial cells, Tregs, and neutrophils in HCC. We speculated that ADNP might facilitate the carcinoma progression and immune invasion by regulating coagulation cascades activity. Furthermore, the outcomes implied that knockdown ADNP could inhibit proliferation, invasion, and migration and lead to effective eradication by accelerating radiation-induced cell death in HCC cells via verification of vitro experiments. Our research first reported that ADNP participated in the proliferation, invasion, migration, immune evasion, and radioresistance in HCC. The findings may aid in developing novel ideas for future research.

Recent years, the bidirectional link between the coagulation system and cancer progression has been established [28]. In cancers, the coagulation cascades likely help to establish the inflammatory TME [29] and facilitate tumor growth, invasion, neo-angiogenesis, and immune evasion [30, 31], contributing to elevating immunotherapy resistance via proteolysis of platelet-bound GARP to activate LTGF-β [32]. Tissue factor, the main initiator of coagulation, plays a critical role in the formation of tumor cell-related microthrombus [33] and coagulation Factor Xa can activate endothelial cells and enhance the cancer cell-endothelium adhesion [34]. Thrombin has been shown to facilitate tumor metastasis in coagulation related manners [35], and direct thrombin inhibitor peptide has been confirmed with inhibitory effects on the invasion and proliferation of tumor cells [36]. Meanwhile, it is intriguing that anticoagulants can consistently improve the efficacy of immune checkpoint inhibitors to enhance anti-tumor immunity [37]. Previous studies have verified the significant relationship between coagulation and tumor immune microenvironment and a coagulation related risk score model was also established to predict prognosis and the response to immunotherapy in HCC [38]. In our study, GSEA analysis indicated the ADNP-related DEGs might regulate the coagulation cascades pathways in HCC. Hence, we speculated ADNP might function as an oncogene in tumor immunosuppression via regulating coagulation cascades pathways especially in HCC. Go enrichment analysis showed ADNP-related DEGs may participate in detoxification of copper ions and the stress response to copper ions related with various signaling pathways and tumor associated biological behaviors [39]. Excess copper ions can lead to cuprotosis, a novel form of cell death resulting in production of ROS on mitochondria, closely related with tumor progression [40]. These results indicated that ADNP maybe a promising biomarker and the concrete mechanism was to be determined.

Immunotherapy, a promising component of oncotherapy, has become the hotspot of cancer research in recent years. As far back as 2009, Manjit et al. discovered ADNP was expressed in T cells, B cells, and monocytes. Additionally, NAP exerted potential immunomodulatory properties via the stimulation of immune cells [41]. In this work, the outcomes of GSEA also showed the significant enrichment of ADNP in the innate immune system. Therefore, it might be an interesting topic to further discuss the correlation between ADNP and antitumor immune response. TME involves immune cells, fibroblasts, endothelial cells, and interacting tumor cells, playing a vital role in cancer proliferation, metastasis, and immune response suppression [42, 43]. It’s reported that MDSCs are involved in cancer cells’ intravasation and regulated immune response by upregulating immune suppressive factors, suppressing T-cell responses, and modulating the cytokine production of macrophages [44–46]. Emerging evidence indicated CAFs participated in the remodeling of the tumor stroma in TME and the regulation of immunocompetent-cell locomotion related to tumor metastasis [47, 48]. Tregs were found to play a potent suppressive role in antitumor immunity within the TME [49]. Another type of innate immune cell, neutrophils promote colorectal cancer metastasis by secreting AGR2 has also been identified [50]. Results in our study revealed that ADNP acted as an oncogene and was significantly positively correlated with CAFs, endothelial cells, MDSC, Tregs, and neutrophils with roles in promoting tumor progression and suppressing antitumor immune effects on HCC. Therefore, we speculated the poor survival and inhibited response to immunotherapy might partly be attributed to upregulated ADNP expression related to these observable increased innate immune cells for HCC patients. In addition, the outcomes also showed a significant negative correlation with immune scores, estimated scores of all analyzed cancers, while a positive correlation with stromal scores merely in LAML. Synthesizing hereinbefore the results, we speculated it might play an immune evasion role in the TME, especially in HCC, and function as the indicator for immunotherapy.

Researchers have illustrated that ADNP exerts dual repercussions, verifying it can accelerate tumor progression or suppress oncogenesis in particular types. In our study, the outcomes unraveled that ADNP was elevated in HCC compared to the normal liver tissues by western blotting and immunochemistry methods. Moreover, the results also represented ADNP expression was positively correlated with a poorer prognosis, higher histologic grade, and progressive pathologic stage in HCC, which was following the previous studies in bladder cancer and HGSOC. In this study, upregulated ADNP expression played a critical role in proliferation, invasion, and migration in HCC cells verified by CCK8 assay, plate clone formation assay, and transwell assay. In addition, A nomogram including significant clinical characteristics and ADNP expression was generated to predict the survival of patients with HCC. This indicated that ADNP is significantly related to tumor development and might be a potential prognostic biomarker for HCC.

Recently, a novel modality, the combination of antiangiogenic drugs plus PD-1 inhibitors and IMRT, was reported to improve the response rate (42.6%) and prolonged the median OS (20.1 months) in HCC [15]. The inspiring results might attribute to the additional anti-angiogenesis drug and radiotherapy for synergistically normalizing tumor vasculature and reprogramming the TME against immune invasion in HCC [51]. However, low immune response and radioresistance in a particular population restricted the clinical application of these novel strategies in HCC. In the enrichment analysis of ADNP-related DEGs in LGG, we also discovered that ADNP might be associated with DNA double-strand break repair, which was essential in radiotherapy resistance. Therefore, we further studied the relationship between ADNP expression and the radiosensitivity of HCC. We confirmed that ADNP was highly expressed in HCC using western blotting and immunochemistry. Then CCK8 assay and IF were performed to explore the impact of knockdown ADNP expression on radiosensitivity in HCC cell lines. The outcomes showed that the enhancement of radiosensitivity was achieved by inhibiting the activity of DNA damage repair after ADNP knockdown. The findings shed light on the potential role of ADNP in acting as a radiotherapy predictor and developing novel ideas for researching into radiotherapy sensitizers in HCC treatment.

In this study, we explored the expression and prognostic value of ADNP across cancers and first reported that ADNP participated in the proliferation, invasion, migration, immune evasion, and radioresistance in HCC. There are also a few limitations in this study. Our results are only based on the bioinformatics analysis and several kinds of functional verification in vitro. Therefore, we plan to establish HCC animal model to verify the results related to radiosensitivity, and explore the molecular mechanism of ADNP in HCC. According to the vitro experiments, we speculated ADNP might be a gene related to cancer stem cells, we will conduct related experiments to verify this conjecture in the future.

## Supplementary Information

**Additional file 1: Supplementary Table 1.** The relationship between ADNP expression and immune infiltration (Stromal score).** Supplementary Table 2.** The relationship between ADNP expression and immune infiltration (Immune score).** Supplementary Table 3.** The relationship between ADNP expression and immune infiltration (Estimate score).

**Additional file 2: Figure S1.** Correlations between ADNP, immune checkpoints and biomarkers. (**A**-**D**)The correlations between ADNP and chemokine, immune inhibitor, immune stimulatorand MHC in multiple cancers. (**E**, **F**) The correlations between ADNP expression andTMB, MSI in cancers.

## Data Availability

The original data presented in the study are encompassed in the article and supplementary material. More interrogations can directly contact the relevant authors.

## Abbreviations

ADNP: Activity-dependent neuroprotective protein
HGSOC: High-grade serous ovarian cancer
COAD: Colon adenocarcinoma
TNBC: Triple-negative breast cancer
GBM: Glioblastoma multiforme
HCC: Hepatocellular carcinoma
TCGA: The cancer genome atlas
GTEx: Genotype tissue expression
HPA: Human Protein Atlas
IHC: Immunohistochemical
OS: Overall survival
DSS: Disease-specific survival
3D: Three-dimensional
KEGG: Kyoto Encyclopedia of Genes and Genomes
GO: Gene Ontology
BPs: Biological processes
MFs: Molecular functions
CCs: Cellular components
BRCA: Breast invasive carcinoma
LGG: Brain lower grade glioma
DEGs: Differentially expressed genes
GSEA: Gene Set Enrichment Analysis
CAFs: Cancer-related fibroblasts
MDSCs: Myeloid-derived suppressor cells
TIMER2.0: Tumor Immune Estimation Resource 2.0
TMB: Tumor mutation burden
MSI: Microsatellite instability
C-index: Concordance index
AUC: Area under the curve
RT-qPCR: Real-time quantitative polymerase chain reaction
CCK-8: Cell counting kit8
IF: Immunofluorescence
CHOL: Cholangiocarcinoma
ESCA: Esophageal carcinoma
STES: Stomach and esophageal carcinoma
LUAD: Lung adenocarcinoma
COADREAD: Colon adenocarcinoma/rectum adenocarcinoma
AML: Acute myeloid leukemia
PAAD: Pancreatic adenocarcinoma
PRAD: Prostate adenocarcinoma
STAD: Stomach adenocarcinoma
SKCM: Skin cutaneous melanoma
HNSC: Head and neck squamous cell carcinoma
WT: Wilms tumor
UCS: Uterine carcinosarcoma
READ: Rectum adenocarcinoma
THCA: Thyroid carcinoma
ALL: Acute lymphoblastic leukemia
OV: Ovarian serous cyst adenocarcinoma
ACC: Adrenocortical carcinoma
UCEC: Uterine corpus endometrial carcinoma
NBL: Neuroblastoma
SARC: Sarcoma
TME: Tumor microenvironment
DCLBCL: Diffuse large B cell lymphoma
LUSC: Lung squamous cell carcinoma
ROC: Receiver operator characteristic
γ-H2AX: Phosphorylated histone H2AX
