# Supplementary material for: ADNP is associated with immune infiltration and radiosensitivity in hepatocellular carcinoma for predicting the prognosis
Source: BMC Med Genomics. 2023 Jul 31;16:178. doi: 10.1186/s12920-023-01592-x (PMC10391866; doi:10.1186/s12920-023-01592-x)
Supplement: Supplementary file 1 — Additional file 1: Supplementary Table 1. The relationship between ADNP expression and immune infiltration (Stromal score). Supplementary Table 2. The relationship between ADNP expression and immune infiltration (Immune score). Supplementary Table 3. The relationship between ADNP expression and immune infiltration (Estimate score). [file 12920_2023_1592_MOESM1_ESM.pdf]

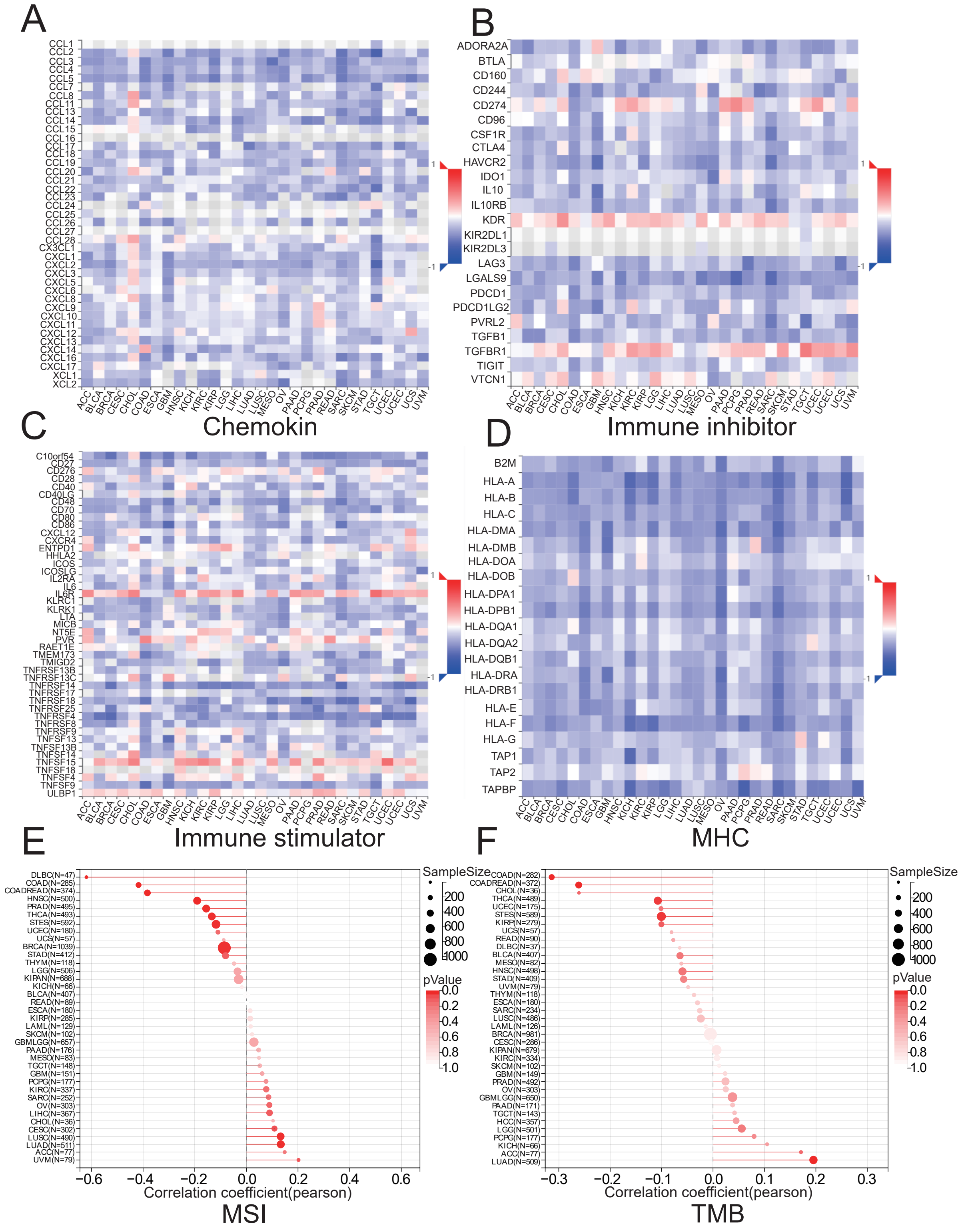
A
CCL1
CCL2
CCL3
CCL4
CCL5
CCL7
CCL8
CCL11
CCL13
CCL14
CCL15
CCL16
CCL17
CCL18
CCL19
CCL20
CCL21
CCL22
CCL23
CCL24
CCL25
CCL26
CCL27
CCL28
CX3CL1
CXCL1
CXCL2
CXCL3
CXCL5
CXCL6
CXCL8
CXCL9
CXCL10
CXCL11
CXCL12
CXCL13
CXCL14
CXCL16
CXCL17
XCL1
XCL2
ACC BLCA BRCA CESC CHOL COAD ESCA GBM HNSC KICH KIRC KIRP LGG LIHC LUAD LUSC MESO OV PAAD PCPG PRAD READ SARC SKCM STAD TGCT UCEC UCEC UCS UVM
Chemokin
1
-1
B
ADORA2A
BTLA
CD160
CD244
CD274
CD96
CSF1R
CTLA4
HAVCR2
IDO1
IL10
IL10RB
KDR
KIR2DL1
KIR2DL3
LAG3
LGALS9
PDCD1
PDCD1LG2
PVRL2
TGFB1
TGFBR1
TIGIT
VTCN1
ACC BLCA BRCA CESC CHOL COAD ESCA GBM HNSC KICH KIRC KIRP LGG LIHC LUAD LUSC MESO OV PAAD PCPG PRAD READ SARC SKCM STAD TGCT UCEC UCEC UCS UVM
Immune inhibitor
1
-1
C
C10orf54
CD27
CD276
CD28
CD40
CD40LG
CD48
CD70
CD80
CD86
CXCL12
CXCR4
ENTPD1
HHLA2
ICOS
ICOSLG
IL2RA
IL6
IL6R
KLRC1
KLRK1
LTA
MICB
NT5E
PVR
RAET1E
TMEM173
TMIGD2
TNFRSF13B
TNFRSF13C
TNFRSF14
TNFRSF17
TNFRSF18
TNFRSF25
TNFRSF4
TNFRSF8
TNFRSF9
TNFSF13
TNFSF13B
TNFSF14
TNFSF15
TNFSF18
TNFSF4
TNFSF9
ULBP1
ACC BLCA BRCA CESC CHOL COAD ESCA GBM HNSC KICH KIRC KIRP LGG LIHC LUAD LUSC MESO OV PAAD PCPG PRAD READ SARC SKCM STAD TGCT UCEC UCEC UCS UVM
Immune stimulator
1
-1
D
B2M
HLA-A
HLA-B
HLA-C
HLA-DMA
HLA-DMB
HLA-DOA
HLA-DOB
HLA-DPA1
HLA-DPB1
HLA-DQA1
HLA-DQA2
HLA-DQB1
HLA-DRA
HLA-DRB1
HLA-E
HLA-F
HLA-G
TAP1
TAP2
TAPBP
ACC BLCA BRCA CESC CHOL COAD ESCA GBM HNSC KICH KIRC KIRP LGG LIHC LUAD LUSC MESO OV PAAD PCPG PRAD READ SARC SKCM STAD TGCT UCEC UCEC UCS UVM
MHC
1
-1
E
DLBC(N=47)
COAD(N=285)
COADREAD(N=374)
HNSC(N=500)
PRAD(N=495)
THCA(N=493)
STES(N=592)
UCEC(N=180)
UCS(N=57)
BRCA(N=1039)
STAD(N=412)
THYM(N=118)
LGG(N=506)
KIPAN(N=688)
KICH(N=66)
BLCA(N=407)
READ(N=89)
ESCA(N=180)
KIRP(N=285)
LAML(N=129)
SKCM(N=102)
GBMLGG(N=657)
PAAD(N=176)
MESO(N=83)
TGCT(N=148)
GBM(N=151)
PCPG(N=177)
KIRC(N=337)
SARC(N=252)
OV(N=303)
LIHC(N=367)
CHOL(N=36)
CESC(N=302)
LUSC(N=490)
LUAD(N=511)
ACC(N=77)
UVM(N=79)
SampleSize
200
400
600
800
1000
pValue
0.0
0.2
0.4
0.6
0.8
1.0
−0.6 −0.4 −0.2 0.0 0.2 0.4 0.6
Correlation coefficient(pearson)
MSI
F
COAD(N=282)
COADREAD(N=372)
CHOL(N=36)
THCA(N=489)
UCEC(N=175)
STES(N=589)
KIRP(N=279)
UCS(N=57)
READ(N=90)
DLBC(N=37)
BLCA(N=407)
MESO(N=82)
HNSC(N=498)
STAD(N=409)
UVM(N=79)
THYM(N=118)
ESCA(N=180)
SARC(N=234)
LUSC(N=486)
LAML(N=126)
BRCA(N=981)
CESC(N=286)
KIPAN(N=679)
KIRC(N=334)
SKCM(N=102)
GBM(N=149)
PRAD(N=492)
OV(N=303)
GBMLGG(N=650)
PAAD(N=171)
TGCT(N=143)
HCC(N=357)
LGG(N=501)
PCPG(N=177)
KICH(N=66)
ACC(N=77)
LUAD(N=509)
SampleSize
200
400
600
800
1000
pValue
0.0
0.2
0.4
0.6
0.8
1.0
−0.3 −0.2 −0.1 0.0 0.1 0.2 0.3
Correlation coefficient(pearson)
TMB

Figure S1. Correlations between ADNP, immune checkpoints and biomarkers. **(A-D)** The correlations between ADNP and chemokine, immune inhibitor, immune stimulator and MHC in multiple cancers. **(E, F)** The correlations between ADNP expression and TMB, MSI in cancers.
