# Supplementary material for: ADNP is associated with immune infiltration and radiosensitivity in hepatocellular carcinoma for predicting the prognosis
Source: BMC Med Genomics. 2023 Jul 31;16:178. doi: 10.1186/s12920-023-01592-x (PMC10391866; doi:10.1186/s12920-023-01592-x)
Supplement: Supplementary file 2 — Additional file 2: Figure S1. Correlations between ADNP, immune checkpoints and biomarkers. (A-D)The correlations between ADNP and chemokine, immune inhibitor, immune stimulatorand MHC in multiple cancers. (E, F) The correlations between ADNP expression andTMB, MSI in cancers. [file 12920_2023_1592_MOESM2_ESM.pdf]

Supplementary Table1 The relationship between ADNP expression and immune infiltration (Stromal score)

Stromal score	r value	p value
TARGET-NB(N=153)	-0.52367	3.76E-12
TCGA-STES(N=569)	-0.27363	3.14E-11
TCGA-GBMLGG(N=656)	-0.23688	8.09E-10
TCGA-LUSC(N=491)	-0.27157	9.50E-10
TCGA-GBM(N=152)	-0.45974	2.55E-09
TCGA-BLCA(N=405)	-0.28284	6.91E-09
TCGA-KIPAN(N=878)	0.176374	1.44E-07
TCGA-SARC(N=258)	-0.28864	2.42E-06
TARGET-LAML(N=142)	-0.37573	4.07E-06
TCGA-STAD(N=388)	-0.2105	2.92E-05
TARGET-WT(N=80)	-0.43469	5.60E-05
TCGA-KIRC(N=528)	0.169308	9.25E-05
TCGA-SKCM-M(N=351)	-0.19689	2.06E-04
TCGA-LGG(N=504)	-0.1542	5.13E-04
TCGA-LUAD(N=500)	-0.1493	8.11E-04
TCGA-KIRP(N=285)	-0.18061	2.21E-03
TCGA-SKCM(N=452)	-0.14171	2.53E-03
TCGA-PCPG(N=177)	-0.22338	2.80E-03
TCGA-THCA(N=503)	-0.11923	7.43E-03
TCGA-LAML(N=214)	0.179555	8.47E-03
TCGA-CESC(N=291)	-0.12589	3.18E-02
TCGA-ACC(N=77)	-0.24168	3.42E-02
TCGA-UCEC(N=178)	-0.15702	3.63E-02
TCGA-BRCA(N=1077)	-0.06343	3.74E-02
TCGA-SKCM-P(N=101)	-0.19918	4.58E-02

Supplementary Table2 The relationship between ADNP expression and immune infiltration (Immune score)

Immune score	r value	p value
TCGA-BRCA(N=1077)	-0.27479	4.12E-20
TCGA-SARC(N=258)	-0.51984	2.91E-19
TCGA-LUSC(N=491)	-0.38316	1.28E-18
TCGA-STES(N=569)	-0.35615	1.86E-18
TCGA-GBM(N=152)	-0.52817	2.69E-12
TCGA-OV(N=416)	-0.33184	3.77E-12
TCGA-GBMLGG(N=656)	-0.25459	3.65E-11
TCGA-SKCM-M(N=351)	-0.34203	4.56E-11
TCGA-THCA(N=503)	-0.28526	7.14E-11
TCGA-LUAD(N=500)	-0.28337	1.10E-10
TCGA-BLCA(N=405)	-0.30886	2.12E-10
TARGET-WT(N=80)	-0.62826	4.42E-10
TCGA-KIRP(N=285)	-0.3466	1.82E-09
TCGA-UCEC(N=178)	-0.42182	4.52E-09
TCGA-SKCM(N=452)	-0.26628	8.94E-09
TCGA-CESC(N=291)	-0.32532	1.34E-08
TCGA-STAD(N=388)	-0.26004	2.04E-07
TCGA-PCPG(N=177)	-0.36595	5.47E-07
TARGET-NB(N=153)	-0.35939	5.06E-06
TCGA-ESCA(N=181)	-0.32785	6.62E-06
TCGA-THYM(N=118)	-0.39609	9.00E-06
TARGET-LAML(N=142)	-0.32431	8.24E-05
TCGA-ACC(N=77)	-0.41852	1.52E-04
TCGA-LGG(N=504)	-0.16456	2.07E-04
TCGA-TGCT(N=132)	-0.30554	3.67E-04
TARGET-ALL(N=86)	-0.36371	5.77E-04
TCGA-SKCM-P(N=101)	-0.32697	8.46E-04
TCGA-MESO(N=85)	-0.34223	1.35E-03
TCGA-COADREAD(N=373)	-0.16237	1.65E-03
TCGA-COAD(N=282)	-0.18051	2.34E-03
TCGA-PRAD(N=495)	-0.13324	2.98E-03
TCGA-UCS(N=56)	-0.37654	4.23E-03
TCGA-LIHC(N=363)	-0.14283	6.41E-03
TCGA-KIRC(N=528)	-0.11202	1.00E-02
TCGA-LAML(N=214)	-0.16754	1.41E-02
TARGET-ALL-R(N=99)	-0.22559	2.48E-02
TCGA-HNSC(N=517)	-0.096	2.91E-02

Supplementary Table3 The relationship between ADNP expression and immune infiltration (Estimate score)

Estimate Score	r value	p value
TCGA-STES (N=569)	-0.34103	5.80E-17
TCGA-LUSC(N=491)	-0.35077	1.16E-15
TCGA-SARC(N=258)	-0.46123	5.38E-15
TCGA-GBM(N=152)	-0.51808	8.16E-12
TCGA-BRCA(N=1077)	-0.2042	1.33E-11
TCGA-GBMLGG(N=656)	-0.2538	4.22E-11
TCGA-BLCA(N=405)	-0.31586	7.81E-11
TARGET-NB(N=153)	-0.46627	1.25E-09
TCGA-SKCM-M(N=351)	-0.31013	2.91E-09
TARGET-WT(N=80)	-0.57736	2.07E-08
TCGA-LUAD(N=500)	-0.2391	6.25E-08
TCGA-THCA(N=503)	-0.23702	7.48E-08
TCGA-STAD(N=388)	-0.25761	2.68E-07
TCGA-KIRP(N=285)	-0.29681	3.32E-07
TCGA-SKCM(N=452)	-0.23669	3.57E-07
TCGA-UCEC(N=178)	-0.34918	1.78E-06
TCGA-OV(N=416)	-0.2291	2.34E-06
TCGA-CESC(N=291)	-0.27109	2.70E-06
TARGET-LAML(N=142)	-0.36637	7.32E-06
TCGA-PCPG(N=177)	-0.31028	2.63E-05
TCGA-LGG(N=504)	-0.16518	1.96E-04
TCGA-TGCT(N=132)	-0.30874	3.16E-04
TCGA-ESCA(N=181)	-0.25444	5.47E-04
TCGA-ACC(N=77)	-0.35913	1.34E-03
TCGA-SKCM-P(N=101)	-0.30499	1.93E-03
TCGA-THYM(N=118)	-0.27552	2.53E-03
TARGET-ALL(N=86)	-0.30857	3.85E-03
TCGA-MESO(N=85)	-0.27653	1.04E-02
TCGA-LIHC(N=363)	-0.13081	1.26E-02
TARGET-ALL-R(N=99)	-0.22238	2.69E-02
TCGA-PRAD(N=495)	-0.08888	4.81E-02
